# Culturally sensitive care for elderly immigrants through ethnic community health workers: design and development of a community based intervention programme in the Netherlands

**DOI:** 10.1186/1471-2458-13-227

**Published:** 2013-03-15

**Authors:** Ilona Verhagen, Wynand JG Ros, Bas Steunenberg, Niek J de Wit

**Affiliations:** 1 Julius Center for Health Sciences and Primary Care, University Medical Center Utrecht, Mailbox 85500, Utrecht, GA, 3508, the Netherlands

**Keywords:** Elderly immigrants, Ethnic minority groups, Community health worker, Culturally sensitive care, Access to health care, Social welfare

## Abstract

**Background:**

In Western countries, health and social welfare facilities are not easily accessible for elderly immigrants and their needs are suboptimally addressed. A transition is needed towards culturally sensitive services to overcome barriers to make cure and care accessible for elderly immigrants. We developed an intervention programme in which ethnic community health workers act as liaisons between immigrant elderly and local health care and social welfare services. In this study we evaluate the effectiveness and the implementation of this intervention programme.

**Methods/design:**

In a quasi experimental design, the effectiveness of introduction of community health workers, health needs assessment, and follow-up intervention programme will be evaluated in three (semi) urban residential areas in the Netherlands and compared with a control group. Community health workers are selected from local ethnic communities and trained for the intervention. Data on health perception, quality of life, and care consumption are collected at baseline and after the intervention programme. Elderly’s informal care givers are included to examine caregiver burden. The primary outcome is use of health care and social welfare facilities by the elderly. Secondary outcomes are quality of life and functional impairments. The target number of participants is 194 immigrant elderly: 97 for the intervention group and 97 for the control group. Implementation of the intervention programme will be examined with focus groups and data registration of community health worker activities.

**Discussion:**

This study can contribute to the improvement of care for elderly immigrants by developing culturally sensitive care whereby they actively participate. To enable a successful transition, proper identification and recruitment of community health workers is required. Taking this into account, the study aims to provide evidence for an approach to improve the care and access to care for elderly immigrants. Once proven effective, the community health worker function can be further integrated into the existing local health care and welfare system.

**Trial registration:**

Trial registration number: ISRCTN89447795

## Background

Generally speaking, elderly immigrants in the Netherlands perceive a poorer health [1-4], more chronic conditions [3,4], and a less favourable mental health compared to native Dutch elderly [3-6]. Moreover, elderly immigrants report more often physical limitations [3,7,8]. Illiteracy [9], poor education [3], low degree of socio-cultural integration [3], poor living conditions [1], and cultural differences in perception of health [1] may contribute to their poorer health status. As a consequence the use of health care facilities differs between elderly immigrants and native Dutch elderly. Immigrant elderly visit their general practitioner more often [1-4,10], whereas the use of physical therapy [2-4,10], home care [3,4,11], and residential care is lower [3,11]. The lower care consumption may be explained by limited knowledge about health care facilities [3,11], language problems [3,4], and/or financial barriers [3,11]. Intercultural differences in perception of health needs and reasons for consultation may also contribute [11,12]. In addition, health care facilities are not easily accessible for elderly immigrants and do not adequately address their needs [13]. One of the major reasons is that elderly immigrants are not involved in the development of services. To improve care and access to care, a transition is needed towards culturally sensitive services whereby elderly immigrants actively participate.

A widely studied means for improving care and access to care for ethnic minorities is the introduction of so called “ethnic community health workers”. These are community health workers who share a common language and are ethnically part of the community. They are trusted and respected by the community members and have an understanding of the community health beliefs and the barriers to health care and social welfare services. They act as intermediaries between community members and providers of services [14,15]. Most community health worker programmes focus on reducing health disparities through improving individual health outcomes on specific areas such as nutrition, diabetes, chronic disease screening, and cancer screening [16]. Research demonstrates that these community health worker programmes are effective in providing health knowledge, increasing health care utilisation, changing health behaviour, and improving health status [17,18]. Little is known about the impact of community advocacy activities by community health workers on access to care and health outcomes [16].

In order to improve care and access to care on a community level, we developed an intervention programme based on the practice of multicultural health brokering [19]. The community health workers serve as cultural health brokers and provide one-on-one support to individuals in gaining access to and navigating the Dutch health and social welfare system. Besides, they catalyse institutional changes. In this way, the community health workers are also engaged in community-level advocacy.

To evaluate the intervention of this present study, a three year study will be conducted to examine the effect of the introduction of community health workers on health perception, quality of life, and health care consumption of elderly immigrants in the Netherlands. The aim of this paper is to describe the design, the content of the intervention, and its strengths and challenges.

## Methods

### Design and setting

We conduct a quasi experiment with a pre-post test design with an intervention group and control group in three (semi) urban residential areas in the Netherlands with each a concentration of immigrants. The intervention consists of selection and training of community health workers, assessment of health needs, and follow-up intervention programme coordinated by the community health workers.

### Participants

Three immigrant populations in the Netherlands with a different migrant background take part: Turks, Moroccans, and Moluccans. These immigrant populations represent a large proportion of the elderly immigrant population in the Netherlands and therefore form a representative reflection of elderly immigrants in the Netherlands.

Turkish and Moroccan men came to the Netherlands as labour migrants in the 1960s and early 1970s. Turkish and Moroccan women moved to the Netherlands in the 1970s and 1980s as a result of family reunification or family formation. The Moluccans, soldiers in the former Dutch colonial army and their families, were “demobilised” to the Netherlands in 1951 after decolonisation of Indonesia (a former Dutch colony) and temporarily housed in Moluccan “camps” due to shortage of housing and the expectation that their stay would be temporary.

### Inclusion criteria

Elderly meeting the following inclusion criteria are eligible to participate:

•Aged 55 years and over

•Living independently (alone or with others)

•Born in Turkey, Morocco, Moluccan Islands or descendant of Moluccan immigrants born in the Netherlands and lived in one of the Moluccan “camps”

### Exclusion criteria

Elderly using care for severe psychiatric disorders are excluded from the study.

### Control group

In order to assess the effectiveness of the intervention, the effects of introduction of the community health worker will be compared with a group where the community health worker is not introduced. No community health worker involvement is provided to the controls. The control group consists of a matched group comparable in size and composition. All controls live outside the three (semi) urban residential areas in the Netherlands that take part in the intervention. We use data from the ongoing Symbol study (Systematic Memory testing Beholding Other Languages) in the Netherlands to collect data on Turkish and Moroccan controls. Recruitment of Moluccan controls will be completed through bilingual interviewers in collaboration with a local social welfare organisation that serves Moluccan elderly.

### Recruitment of intervention participants

Intervention participants are identified, recruited, and selected by community health workers representing the elderly immigrant population. All community health workers have strong ties to and rootedness within the local migrant community. All elderly reached by the community health worker will be screened on the in- and exclusion criteria. Eligible elderly receive study information and an informed consent form in the preferred language for participation. If language problems make reading impossible, the community health worker reads the information to the potential participant. If the participant is not willing and/or not able to sign the written informed consent form, the community health worker holds the option of offering the potential participant the possibility for oral consentform. After permission, the community health worker makes an appointment for the baseline interview. In case of an informal care giver, the care giver is also invited by the community health worker to participate to measure care giver burden.

The community health workers do not conduct the interviews to avoid bias in the data collection. The interviews will be conducted by bilingual interviewers who are not involved in intervention activities. To ensure the examination of the intervention under real world circumstances no (financial) incentives will be offered for study participation.

### Intervention programme

The intervention consists of four steps:

1. In the first step, the community health worker conducts home visits to the elderly to examine health problems, barriers to health care and social welfare services, and needs for adequate care. During the home visits, the community health worker registers the outcomes. The community health worker provides information on health and social welfare and refers to health and social welfare services if desirable. In addition to the home visits, information meetings are set up by the community health worker in collaboration with local migrant organisations.

2. In the second step, the community health worker identifies commonly shared problems based on the home visits and organises problem focused working groups of eight to twelve elderly persons (elderly who experience one of these problems), their family members/informal care givers, and local providers of health care and social welfare facilities.

3. In the third step, the community health worker co-operates with the elderly and providers of health care and social welfare services in finding solutions and in creating and conducting improvement programmes. These consist of concrete initiatives necessary for providing care and social welfare services that meet the health and social welfare needs of the elderly involved.

4. In the fourth step, these new initiatives will be implemented by the local providers of health care and social welfare facilities in their existing health care and welfare services in collaboration with the elderly. The community health worker monitors the process of implementation of the improvement programmes in the community.

Besides, the community health worker serves as participator in the research process by approaching participants for interviews.

### Selection of community health workers

The community health worker will be paid as a part-time worker who is connected to, but not a staff member of one of the local social welfare organisations involved to keep their independent role as intermediary.

A local programme coordinator will preselect community members who have the qualities and skills required to become a community health worker:

•Empathetic attitude towards elderly

•Understanding of elderly’s needs and socio-cultural norms

•Known within the community as a trusted and respected community member

•Ability to communicate with representatives of the elderly involved and providers of health care and social welfare services

•Ability to provide community outreach through offering information meetings on health care and social welfare

•Providing advice and referring elderly in need to health care and social welfare services

•Knowledge of local health care and social welfare facilities

•Knowledge of the Dutch care and social welfare system

### Training and supervision of community health workers

The community health worker will be trained and prepared for his/her roles and tasks. Trainers with extensive experience in training community health workers collaborate with the research group in developing and carrying out the training. The training consists of two, six hour sessions. During the sessions, the trainers explain and discuss the intervention programme. The sessions have an interactive character and contain exercises to practice necessary skills and a role play with an actor. Furthermore, the research team will discuss the research process and the collaboration between the community health workers and the research team.

During the intervention programme, ongoing supervision will be delivered by the local programme coordinator acting as a mentor and supervisor. In addition, the community health workers participate on a regular basis in intervision sessions by the trainers involved in the training sessions to address difficulties and to further develop skills. The research team can be contacted for advice in case of questions and/or difficulties in the research activities.

### Assessments

Data are collected at two points in time. A baseline assessment within two weeks after the home visit by the community health worker (T0) and a follow-up assessment 18 months after the baseline assessment (T1). The assessments are structured face-to-face interviews in the preferred language of the respondent performed by trained bilingual interviewers. To ensure the compatibility of interviews across the different interviewers and to minimise the variation in results, the interviewer will use standardised, translated versions of the questionnaire instead of translating the questionnaire during the interview. The interviewers have a Turkish, Moroccan or Moluccan background.

All interviewers will receive a training that consists of three hours. During this training, the content of the study and the collaboration between the interviewers and the research team will be explained and discussed. An instruction on the translated questionnaires will be given. The training is interactive and contains a role play to address possible difficulties. All interviewers will further receive interview guidelines and a definition list of medical terms used in the questionnaire. During the period of data collection, the research team can be contacted for advice in case of questions and/or difficulties.

To ensure the quality of the collected data the research team monitors the questionnaires on completeness and occurrence of impossible answers.

### Outcomes

The primary outcome measure is the use of health care and social welfare facilities which will be assessed by self-reported care consumption.

The secondary outcome measures are perceived quality of life and functional impairments in daily functioning. Perceived quality of life will be measured by using the validated Short Form-12 (SF-12) [20] and the EQ-5D + C [21]. Functional impairments will be measured by using the Katz-15 [22] frequently used to assess functional status of elderly and valid to assess functional ability of Turkish, Moroccan, and native Dutch elderly [23].

### Moderating variables

Several possible moderators of care consumption, quality of life, and functional impairments will be explored:

1. Multimorbidity will be assessed by self-reported illnesses and conditions.

2. Anxiety and depressive disorders will be screened by the Kessler Psychological Distress Scale (K10) [24], a reliable and valid measure for screening anxiety and depression among Turkish, Moroccan, and native Dutch subjects [25].

3. Loneliness will be assessed by using the Jong Gierveld loneliness scale [26,27], a sufficiently reliable instrument for measuring loneliness among elderly [28] and used in studies among older people in the Netherlands [29].

4. Acculturation will be measured by using the adapted Psychological Acculturation Scale (PAS) [30], a reliable instrument used among Moroccan adults [30]. The PAS is originally designed by Tropp et al. [31] and used among Anglo Americans and Latino/Hispanic Americans.

5. Feelings of loss as part of acculturation will be measured by the subscale “loss” from the Lowlands Acculturation Scale (LAS) [32], a valid measure used among Turkish and Moroccan migrants, and other migrant populations in the Netherlands [32-36].

6. Dutch language proficiency will be assessed by self-reported difficulty in speaking Dutch.

### Additional data collection

Socio-demographic factors such as age, sex, marital status, ethnicity, migration background, living situation, religiosity, and educational level will be obtained at baseline.

### Translation of instruments

The interviewers will use translated versions of the instruments for the Turkish, Moroccan-Arabic, Berber or Malay speaking participants. Several translated instruments used in other studies will be used:

•Turkish version of the K10 used in a study by Fassaert [37]

•Turkish, Moroccan-Arabic, and Berber version of the Jong Gierveld loneliness scale used in an ongoing study by Uysal-Bozkir et al. [38]

•Turkish and Moroccan-Arabic version of the SF-12 used in a study by Denktaş [8]

•Moroccan-Arabic version of the adapted PAS used in a study by Stevens [39]

For the other instruments complete translated versions are accomplished using a forward-backward procedure [40]. Two forward translators, both native speakers, translated the instruments from the Dutch version into the target language (Turkish, Berber, Moroccan-Arabic, and Malay) and were translated back into Dutch by a backward translator, a native speaker of Dutch and fluent in the target language. Differences from the Dutch version were discussed with the translators and resolved.

### Sample size calculation

No comparable interventions studies are available to determine the sample size. Therefore, we conducted a power analysis to determine the number of elderly based on a theoretical effect size assessed effect size. Based on research literature an effect size of 0.50 is estimated to provide a minimal clinically important difference [41]. We chose a moderate theoretical effect size of 0.40 to ensure that potential relevant findings are included. Power analysis showed that 97 elderly in each group should be included (an effect size of 0.40, an alpha of 0.05 and a beta of 0.20). Thus, we need a total of 194 elderly.

### Process evaluation

Each part of the intervention delivered by the community health workers will be monitored and registered. This qualitative data will be gathered from registration forms. In the end, focus groups will be carried out with the elderly and their informal care givers, the community health workers, and the stakeholders in the (semi) urban residential areas.

### Data analysis

The data will be analysed using SPSS version 20. We compare the use of health care and social welfare facilities, quality of life, and functional impairments between the intervention group and the control group. To correct for the clustering of participants within wards and for baseline characteristics, multilevel analysis will be used to evaluate differences in outcome between the two groups regarding baseline and follow up measurements. Estimates will be performed with 95% confidence intervals. An intention-to-treat analysis will be conducted consisting of data from all subjects including those lost to follow up. Audio recordings of the focus groups will be transcribed verbatim and analysed thematically using NVivo version 9.

### Ethical principles

Participants are informed that participation is voluntary and anonymity is guaranteed. Besides, participants are informed that their participation could be finished during the study without giving a reason and without negative consequences. Participants sign an informed consent form. Those who are not willing and/or not able to sign the written informed consent form give oral consent.

We submitted our study protocol to the Medical Ethics Committee of the University Medical Center Utrecht (UMCU) if this study falls under the Medical Research Involving Human Subjects Act (WMO). The committee judged that our study does not meet the WMO criteria and therefore is not subject to the WMO.

## Discussion

In the development of this study we made a number of choices that need to be addressed. We chose a quasi experiment, because a randomised controlled trial is less suitable for community-based intervention research using community health workers [42]. A randomised controlled trial whereby the community health workers randomise participants to specific conditions is difficult to translate into practice, because this may conflict with their role as a community health advocate due to the assignment to control conditions with no direct benefit to the community members they serve [42].

For the generalisability of our findings, we chose for diversity in immigrant populations (migrant background) and sites (semi large urban residential areas) and we recruited several community health workers on each site to deliver the intervention.

To ensure a representative reflection of elderly immigrants in the Netherlands, the three immigrant populations in this study were chosen, because these together represent the majority of the elderly immigrant population in the Netherlands. The three (semi) urban residential areas in the Netherlands were selected, because of the concentration of the immigrant populations involved in this study. The diversity in immigrant populations and the enrolment of multiple community health workers provide the opportunity to compare the results between three different locations with each a specific concentration of migrants and to examine if and under which conditions the community health worker have impact on the outcome measures.

The outcomes measures in this study were chosen to ensure that the effectiveness of the intervention is assessed on outcomes that are directly relevant for elderly immigrants. The health problems among elderly immigrants often lead to long-lasting functional limitations and need for health care [23]. Functional limitations and perceived health may have impact on their daily functioning and quality of life [3].

### Strengths

A positive aspect of the present study is that the recruitment of the participants is conducted by community health workers who are trusted and respected members of the communities and ethnically matched with the elderly immigrants involved. Regarding to other studies, this is mentioned as a successful strategy for recruitment in ethnically diverse populations [18].

Additional, the interviews are conducted by well-trained bilingual interviewers of the same ethnicity. In combination with translated instruments in the native language of the elderly involved we expect that this will result in the participation of elderly who are mostly excluded from research due to language barriers, illiteracy, and mistrust of research.

The design of a multi-site, quasi experimental design enables us to examine the effectiveness of the intervention among elderly from different immigrant groups compared to elderly with a similar ethnic background in a middle and large urban setting. Moreover, the study population will be recruited from different (semi) urban residential areas and varied immigrant populations and the intervention involves multiple community health workers in the delivery. These enhance the external validity of the intervention and make our findings generalisable to immigrant populations that represent a large proportion of the elderly immigrant population in the Netherlands.

Data for this study will be collected using mixed methods including quantitative data and qualitative data. This enables us to examine the effectiveness of the intervention with standardised instruments and to conduct a process evaluation on the delivery of the intervention programme with focus groups and analysing data registration forms. Moreover, this triangulation enhances the reliability of our results.

### Limitations

Besides the strengths, there are some limitations to this study. Health and health care use will be measured by self-report. Although, self-reported measures may influence the estimate of care utilisation, self-reporting is considered a reasonably valid estimation of ethnic differences in use of health care [43]. At the same time, however, not all measures in this study have been cross-cultural validated yet.

### Challenges

First of all, it is important that the immigrant elderly reached by the community health workers also include the more difficult-to-reach frail elderly. Therefore, only community health workers with deep roots and strong ties within the local migrant community will be selected.

The intervention fidelity is of crucial importance too. To avoid variation in the intervention delivery, each community health worker will be trained in all of the components of the intervention programme and receive ongoing supervision during the intervention delivery. Besides, documentation from the community health workers regarding their intervention activities will be reviewed. Ongoing feedback to community health workers regarding the intervention delivery further enhances the intervention fidelity.

Additional, proper identification and recruitment of the community health workers are crucial for a successful implementation of the intervention in this study. Therefore, the community health workers will be identified and recruited by using a profile consisting of necessary qualities and skills.

Finally, commitment of local community based health care or social welfare organisations is needed to start up culturally sensitive care and integrate this care into their existing services. The required commitment will be obtained by actively involving these organisations in the preparation of the study and formally establish the corporation by means of a corporation agreement.

### Study status

Baseline data collection has been accomplished in July 2012. The follow up data collection will be completed in December 2013. The study results will be expected in spring 2014.

### Description of risks

No risks are related to this study.

## Competing interests

The authors declare that they have no competing interests.

## Authors’ contributions

IV drafted the manuscript. BS, WR, and NW designed the study and wrote the grant application. All authors are actively involved in the study and approved the final draft of the manuscript.

## Pre-publication history

The pre-publication history for this paper can be accessed here:

http://www.biomedcentral.com/1471-2458/13/227/prepub
